# Seroprevalence of human immunodeficiency virus, hepatitis B and C viruses among haemodialysis patients in two newly opened centres in Cameroon

**DOI:** 10.11604/pamj.2017.27.235.13121

**Published:** 2017-07-31

**Authors:** Henry Namme Luma, Marie Patrice Halle, Servais Albert Fiacre Bagnaka Eloumou, Fondong Azingala, Felicite Kamdem, Olivier Donfack-Sontsa, Gloria Ashuntantang

**Affiliations:** 1 Internal Medicine Service, Douala General Hospital, Douala, Cameroon; 2 Faculty of Medicine and Biomedical Sciences, University of Yaoundé, Yaoundé, Cameroon; 3 Faculty of Medicine and Pharmaceutical Sciences, University of Douala, Douala, Cameroon; 4 Faculty of Health Sciences, University of Buea, Cameroon

**Keywords:** Haemodialysis, hepatitis B, hepatitis C, HIV, Prevalence

## Abstract

**Introduction:**

Haemodialysis (HD) patients are potentially susceptible to infection with blood borne viral agents especially; Human Immunodeficiency Virus (HIV), hepatitis B (HBV) and hepatitis C Viruses (HCV), compared to the general population. We described their epidemiology in two newly created haemodialysis units in Cameroon: the Buea and Bamenda haemodialysis centres.

**Methods:**

A cross sectional study: included were patients who had spent at least three months in haemodialysis. HBV, HCV and HIV serologies were determined and patients' characteristics extracted from patient's records.

**Results:**

We included 104 participants (44 in Buea and 60 in Bamenda). Mean age was 48 years and 65.4% were men. Median duration in dialysis was 14 months. One of the three viral markers was present in 40.1% of patients. The hepatitis B surface antigen, anti-HCV and anti-HIV antibody prevalence were respectively 10.6%, 19.2%, 13.5%. A history of sexually transmitted infection was the only variable associated with anti-HIV antibody positivity

**Conclusion:**

The sero-prevalence of HBsAg, HCV and HIV are high in the two centres. HIV prevalence may have reflected its etiology as a cause of ESKD. HCV remains a cause for concern and needs further evaluation. There is urgent need for the implementation of preventive and control measures.

## Introduction

Hepatitis B Virus (HBV), Hepatitis C Virus (HCV) and Human Immunodeficiency Virus (HIV) infections are major public health problems worldwide. It is estimated that over 350 million people are chronically infected with HBV [1], more than 185 million with HCV [2] and over 35 million with HIV [3]. The greatest burden of disease is in low and middle income countries. These viruses singly or as co-infections can induce hepatic inflammation which potentially results in progressive fibrosis leading to cirrhosis and hepatocellular carcinoma. Haemodialysis (HD) patients are potentially susceptible to infection with these blood borne viral agents (HBV, HCV, HIV) compared to the general population making them a persistent public health concern as they are a cause of increased morbidity and mortality [4, 5]. The prevalence of HBV in dialysis facilities in Western Europe, United States of America and Japan has been shown to range between 0-6.6% [6, 7]. By contrast in Asia Pacific it ranged from 1.3 to 14.6% [8]. The prevalence of HCV in HD varies greatly ranging from a low 1% to as high as 70% being generally below 5% in high income countries [9].

The major reasons for this high prevalence of infection with HCV and HBV in HD patients are; high prevalence of infection in the general population, lack of standard methods of prevention and effective vaccination, inadequate disinfection of dialysis machines and other medical equipment as well as the spread of infection from one patient to another [5]. In Cameroon, with a population of over 24 million inhabitants [10], there were only two HD centres located in tertiary hospitals in the two largest cities, Yaoundé and Douala. To address the growing demand for management of End stage Kidney Disease (ESKD), HD services were progressively decentralised from 2009 to second level regional hospitals. There are now a total of seven centres. In the Douala HD centre, the largest and busiest in Cameroon, the prevalence of HBV and HIV in 166 HD patients was 7.8% and 10.8% respectively [11]. However, in this same centre and at around the same time frame, baseline prevalence of HBV, HCV and HIV in ESKD patients prior to commencing HD was 6.2%, 20.6% and 9.3% respectively [12]. Focus on the prevalence of blood borne viruses in HD centres and the determination of their risk factors will enable health planners in Cameroon to operate more effectively, to reduce disease prevalence and ultimately reduce rates of morbidity and mortality. Initial and regular evaluation may play an important role especially in the new centres which have limited experience with HD. The aim of this study was to determine the prevalence and factors associated with HBsAg, anti-HCV and HIV antibodies among HD patients in two newly created HD centers in Cameroon.

## Methods

### Study design and setting

**This was a cross-sectional study carried out from the 1^st^ of February to the 30^th^ of April 2013 in the Buea and Bamenda HD Centres:** The Buea HD centre was opened in 2011 and is located in the Buea regional hospital. It serves the inhabitants of the South West region. This centre is equipped with eight HD generators. The staff include two general practitioners (qualified medical doctors with no further postgraduate or specialist qualification) who underwent a five months training course on HD, eight nurses, four of whom were HD nurses, one trained technician and two cleaners. Two dialysis sessions take place every day from Monday to Saturday, with each session lasting averagely 4 hours. There are four nurses who work throughout both sessions and rest after each day. The Bamenda HD centre opened in 2009 and offers treatment to the inhabitants of two regions; the North West and West regions. It is equipped with 7 HD machines. The staff is made up of one trained general practitioner, seven nurses, four of whom were HD nurses, one trained technician and two cleaners. This center operates from mondays through saturdays and three HD sessions take place each day. Three nurses work for the first two shifts and are replaced by 2 nurses who take the last shift. The HD machines are Fresenius^®^ 4008S generators (Fresenius Medical Care, Hamburg, Germany), using synthetic polysulfone dialysis membrane and bicarbonate dialysate. Chemical disinfection of HD generators is carried out between sessions in accordance with the manufacturer's protocol. No dialyser reuse and no isolation policy for HCV, HBV or HIV infected patients are practiced in both centres. Heparin in multi-dose vials is the main anticoagulant and recombinant erythropoietin in prefilled syringes is available for patients who can afford it. Patients on maintenance HD as treatment undergo two 4-hour dialysis sessions a week. Each hospital has a blood bank and blood donors are routinely screened for HIV, HBV, and HCV. These two centres are supervised by a nephrologist from the Yaounde General hospital HD Centre. Yaounde is over 300km from each of the centres.

**Study population patients**: In both centres, all patients on maintenance hemodialysis for at least three months were eligible for this study. Informed and written consent was obtained from each participant. Data was collected, using a pre-tested structured questionnaire, by a trained final year medical student. Socio-demographic data (age, residence, marital status and educational level), potential risk factors for acquiring blood borne infections (blood transfusions, previous surgery, tattoos, history of sexually transmissible infections (STI) and duration on HD) were noted and patient files were used to complete the information and clinical data. Laboratory procedures; five millilitres of blood was collected by a nurse using standard procedures for peripheral venipuncture from each patient before commencement of the HD session. This was to avoid interaction of the blood with heparin which is given during dialysis. We screened for HIV using immunochromatographic tests (Determine HIV 1/2, Alere Medical Co Chiba, Japan) and then immuno-enzymatic assay (Immunocomb II HIV I and II BiSpot, Orgenics, Yavne, Israel) for confirmation and differentiation. Serum samples were obtained by centrifugation of blood samples at 3,000 g for 10 minutes then stored at-20°C in the hospital laboratories before being transported to the Douala General Hospital Laboratory at the end of the week for analysis of HBsAg and Anti HCV antibody. Serological testing for HBsAg and anti-HCV antibodies were done using enzyme immunoassay technique (EIA) Enzygnost 6.0.Berhing for HBsAg and Anti HCV 4.0 respectively (Siemens Healthcare Diagnostics product GmbH Marburg, Germany). Ethical clearance was sought and obtained by the institutional review board (IRB) of the Faculty of Health Sciences, Buea. Administrative clearance was also given by the South West and North West Regional delegates of public health and the chief medical officers for the Buea and Bamenda regional hospitals.

**Statistical analysis**: Results are presented as count (percent). We categorised age in groups of 20 years and used the Chi^2^ or fishers exact test to look for association between HbsAg, anti-HCV and anti-HIV antibodies and other categorical variables. We also used a 2 sample proportion test to compare the prevalences of HbsAg, anti-HCV and anti-HIV antibodies in the two regions. The level of significance in hypothesis testing was set at the level of 5%. Results were analysed using STATA 12 (Stata Corporation, college station, Texas, USA).

**Ethical considerations**: Ethical clearance was obtained from the institutional review board of the Faculty of Health sciences, University of Buea. Administrative authorization was sought and obtained from the South West and North West Regional delegates of public health and the chief medical officers for the Buea and Bamenda regional hospitals. Confidentiality, anonymity and privacy of all participants were guaranteed at all levels of this study. Written consent was provided by each and every participant.

## Results

**Figure 1:**
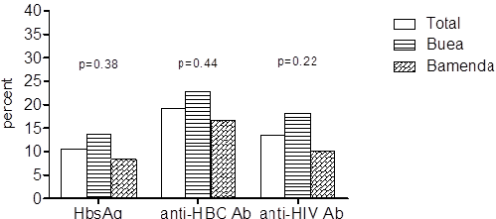
HbsAg, anti-HCV and anti-HIV antibodies positivity by region; P values compare the prevalences in the 2 regions (Bamenda vs Buea)

We included 104 participants in this study (44 in Buea and 60 in Bamenda) and their mean age was 48 (sd: 16) years. Table 1 depicts the baseline characteristics of the study population. The main cause of ESKD was hypertension in 40.4% (42/104) of our study population, followed by chronic glomerulonephritis, 19.2% (20/104). Other causes were: HIV associated nephropathy (HIVAN) 11.5% (12/104); diabetes, 7.7% (8/104); obstructive nephropathy, 2.9% (3/104). The cause was unknown in 13.5% (14/104) of cases. The median duration on HD was 14 months (Interquartile range (IQR):5.5-32). The total number of patients who had at least one of the three viral markers was 40.1% (42/104).The prevalence of HBsAg was 10.6% (11/104), from which 13.6% (6/44) in Buea and 8.3% (5/60) in Bamenda. The prevalence of anti-HCV antibodies was 19.2% (20/104), with 22.7% (10/44) in Buea and 16.7% (10/60) in Bamenda. The prevalence of anti-HIV antibodies was 13.5% (14/104) with 18.2% (8/44) in Buea and 10.0% (6/60) in Bamenda (Figure 1). There was no statistically significant difference in the prevalence of viral markers between the two dialysis centres (Figure 1). Two HD patients had co-infection with HIV/HBsAg giving a prevalence of 1.9% while 1 patient had HIV/ anti-HCV co-infection. Table 2 shows the prevalences of HbsAg, anti-HCV and anti-HIV antibodies by sociodemographic, comorbidities and potential risk factors for acquiring these infections in the participants. Diabetes was associated with anti-HCV antibody positivity, whilst age and history of STI were both associated to anti-HIV antibody positivity. No association was found between HBsAg positivity and all the variables tested.

**Table 1:** Baseline characteristics of the study population (N=104)

Characteristics	N(%)	Characteristics	N(%)
**Age**		**Hypertension**	
<30	14(13.5)	No	16(15.4)
30-49	41(39.4)	Yes	88(84.6)
50-69	39(37.5)	**Diabetes**	
≥70	10(9.6)	No	83(79.8)
**Gender**		Yes	21(20.2)
Male	68(65.4)	**History of STI**	
Female	36(34.6)	No	42(40.4)
**Education**		Yes	62(59.6)
Illiterate	2(1.9)	**Blood transfusion**	
Primary	32(30.7)	No	5(4.8)
Secondary	37(35.6)	Yes	99(95.2)
Tertiary	33(31.7)	**Surgical intervention**	
**Duration in haemodyalisis**		No	82(78.8)
<24 months	64(61.5)	Yes	22(21.2)
>24 months	40(38.5)	**Scarification**	
**Alcohol**		No	9(8.7)
No	75(72.1)	Yes	95(91.3)
Yes	29(27.9)		

**Table 2:** Prevalence of HbsAg, anti-HCV and anti-HIV antibodies by characteristics of study participants

Characteristics	Total	HbsAg			Anti-HCV antibodies			Anti-HIVantibodies		
		Negative	Positive	P	Negative	Positive	P	Negative	Positive	P
**Age**										
<30	14(13.5)	12(85.7)	2(14.3)		10(71.4)	4(28.6)		14(100.0)	0(0.0)	
30-49	41(39.4)	33(80.5)	8(19.5)	0.12	38(92.7)	3(7.3)	0.10	30(73.2)	11(26.8)	0.03
50-69	39(37.5)	38(97.4)	1(2.6)		30(76.9)	9(23.1)		36(92.3)	3(7.7)	
≥70	10(9.6)	10(100.0)	0(0.0)		6(60.0)	4(40.0)		10(100.0)	0(0.0)	
**Gender**										
Male	68(65.4)	62(91.2)	6(8.8)		52(76.5)	16(23.5)		61(89.7)	7(10.3)	
Female	36(34.6)	31(86.1)	5(13.9)	0.51	32(88.8)	4(11.1)	0.19	29(80.6)	7(19.4)	0.23
**Education**										
Illiterate	2(1.9)	2(100.0)	0(0.0)		1(50.0)	1(50.0)		0(0.0)	2(100.0)	
Primary	32(30.7)	29(90.6)	3(9.4)		28(87.5)	4(12.5)		30(93.7)	2(6.3)	
Secondary	37(35.6)	31(83.8)	6(16.2)	0.53	30(81.1)	7(18.9)	0.35	31(83.7)	6(16.3)	0.02
Tertiary	33(31.7)	31(93.9)	2(6.1)		25(75.8)	8(24.2)		29(87.8)	4(12.2)	
**Duration in HD**										
<24 months	64(61.5)	55(85.9)	9(14.1)		54(84.5)	10(15.6)		54(84.4)	10(15.6)	
>24 months	40(38.5)	38(95.0)	2(5.0)	0.20	30(75.0)	10(25.0)	0.56	36(90.0)	4(10.0)	0.56
**Alcohol**										
No	75(72.1)	66(88.0)	8(12.0)		60(80.0)	15(20.0)		64(85.3)	11(14.7)	
Yes	29(27.9)	26(89.6)	3(10.4)	>0.99	24(82.7)	6(17.3)	0.79	26(89.6)	3(10.4)	0.75
**Hypertension**										
No	16(15.4)	16(100.0)	0(0.0)		13(81.3)	3(18.7)		13(81.3)	3(18.7)	
Yes	88(84.6)	77(87.5)	11(12.5)	0.21	71(80.7)	17(19.3)	>0.99	77(87.5)	11(12.5)	0.45
**Diabetes**										
No	83(79.8)	72(86.7)	11(13.3)		71(85.5)	12(14.5)		72(86.7)	11(13.3)	
Yes	21(20.2)	21(100.0)	0(0.0)	0.12	13(61.9)	8(38.1)	0.03	18(85.7)	3(14.3)	>0.99
**History of STI**										
No	42(40.4)	36(85.7)	6(14.3)		35(83.3)	7(16.7)		42(100.0)	0(0.0)	
Yes	62(59.6)	57(91.9)	5(8.1)	0.35	49(79.0)	13(21.0)	0.62	48(77.4)	14(22.6)	0.001
**Blood transfusion**										
No	5(4.8)	4(80.0)	1(20.0)		4(80.0)	1(20.0)		4(80.0)	1(10.0)	
Yes	99(95.2)	88(88.8)	10(11.2)	0.44	80(80.8)	19(19.2)	>0.99	86(86.9)	13(13.1)	0.52
**Surgical intervention**										
No	82(78.8)	72(87.8)	10(12.2)		66(80.5)	16(19.5)		19(86.4)	3(13.6)	
Yes	22(21.2)	21(95.5)	1(4.5)	0.90	18(81.8)	4(18.2)	0.45	71(86.6)	11(13.4)	>0.99
**Scarification**										
No	9(8.7)	9(100.0)	0(0.0)		6(66.7)	3(33.3)		8(88.9)	1(11.1)	
Yes	95(91.3)	84(88.4)	11(11.6)	0.59	78(82.1)	17(17.9)	0.37	82(86.3)	13(13.7)	>0.99

## Discussion

The HD procedure per se, as well as disturbances in both innate and adaptive immunity significantly contributes to increased susceptibility to infections in patients on maintenance HD. Among these are blood borne viral infections (HBV, HCV, HIV) which are a major cause of morbidity and mortality [13]. Forty percent of our study population had at least one of the three viral markers. Due to the nature of the HD procedure this raises the issue of safety concerning the spread of these infections among HD patients and the staff of the unit [13]. The challenges of infection control standards for the prevention of these infections need to be identified especially in new centres with limited experience in HD. Compared to the Douala centre with over 25 years of HD experience, the two new centres in Buea and Bamenda had been functioning for two and four years respectively. The medical and nursing staff had received very little and makeshift training. It is therefore imperative that infection control policies be reinforced by health care authorities. Kidney disease improving global outcomes (KDIGO) clinical practice guidelines for preventionof hepatitis in HD shows strong quality of evidence that HD units should ensure implementation of and adherence to strict infection control procedures designed to prevent transmission of blood borne pathogens [14]. Infection control procedures should include hygienic precautions that effectively prevent the transfer of blood or fluids contaminated with blood-between patients, either directly or via contaminated equipment or surfaces [14].

The prevalence of each viral marker was higher in the newer Buea centre than in Bamenda. However, this difference was not statistically significant. The prevalence of a positive HBsAg in our study population was 10.6%. This is higher than in the Douala centre which is the biggest in Cameroon, where the prevalence was 7.8% of 166 patients. In spite of that, both fall within the range of that in the general population (8-12%). This may therefore just be a reflection of the prevalence of HBV in the general population in Cameroon (Halle 2013 HSD). HBV prevalence was found to be lower in HD centres in Libya, Iran, Sudan and Palestine ranging from 2.6 to 4.5% [5, 15–17]. Globally, the incidence and prevalence of HBV in HD centres has dropped markedly as a result of isolation strategy for HBsAg positive patients, the implementation of infection control measures and introduction of HBV vaccination [18] all of which needs to befully assessed in our setting. We found the prevalence of anti HCV antibodies at 19.2% to be much higher than that of the general population (13.8%). This finding is surprisingly similar to the baseline prevalence of predialysis ESRD patients in Douala, which was 20.6% of 97 patients [12]. Even in low HCV burden countries, anti HCV antibody positivity have been found to be much higher than that of the general population [5, 15–17, 19, 20]. In most of these studies the frequency of blood transfusion and longer duration on HD were found to be associated with acquisition of anti HCV antibody positivity. We did not find any of these associations.

However, one would have expected that with more efficient screening for blood borne viruses in donated blood nowadays, transmission by this route would have been less likely. This may mean that we need to pay more attention to prevent nosocomial transmission [7, 14]. We found Diabetes Mellitus to be associated with HCV positivity. This has been described by other authors [7, 21, 22]. The prevalence of HIV in this study was almost three times above the national prevalence which is at 4.5% [23]. This finding was similar to that found in Douala. In two studies in Iran [5, 20] and one in Morocco [24], there was no HIV positivity in any of the patients in the HD centres. HIVAN shows a predilection for the black race accounting for about 65% of the ESKD among HIV positive patients [13, 25–27]. HIV was the cause of ESKD in 11.5% of our total study population. This high HIV prevalence thus reflects both the burden of HIV in our setting and also as a cause of ESKD. The association between HIV positivity and STI as shown in this study could be a reflection of the latter as a risk factor for acquiring HIV. There were some limitations in this study. Firstly, the sample size was small. However, patients from two HD centres were studied in comparison to other single centre studies. As in all cross-sectional studies this one cannot permit us to determine whether the patients acquired the infection before or after commencement of HD. Our screening methods cannot adequately exclude HBV infection because of the increased prevalence of occult infections in HD [20] and for HCV infection, anti HCV positivity was not accompanied by HCV-RNA testing. However, this study provides a general overview of the prevalence of three blood borne viral infections in two newly opened HD centres in Cameroon.

## Conclusion

The sero-prevalence of HBsAg, HCV and HIV are high in the two newly opened HD centres studied in Cameroon. While the HBV prevalence in chronic HD patients may have reflected the burden of the disease in the general population, that of HIV may have reflected its etiology as a cause of ESKD. HCV in HD remains a cause for concern and needs to be further evaluated. There is need for multicentre studies in Cameroon as it will give a better picture of the burden of these blood borne viral infections in HD. This improved understanding will help to emphasize the urgent need for the implementation of preventive and control measures that will help reduce infections, thereby improving the quality of life of HD patients.

### What is known about this topic

- The greatest burden of HBV, HCV and HIV is in low and middle income countries;
- Haemodialysis (HD) patients are potentially susceptible to infection with blood borne viral agents (HBV, HCV, HIV) compared to the general population;
- In addition to addressing the growing demand for management of End stage Kidney Disease (ESKD) in this setting, these viral agents are an additional cause of increased morbidity and mortality.

### What this study adds

- The sero-prevalence of HBsAg, HCV and HIV are high in the two newly opened HD centres studied in Cameroon as forty percent of our study population had at least one of the three viral markers;
- While the HBV prevalence in chronic HD patients may have reflected the burden of the disease in the general population, that of HIV may have reflected both its burden in the general population but also its etiology as a cause of ESKD;
- The association between HIV positivity and STI as shown in this study could be a reflection of the latter as a risk factor for acquiring HIV.

## Competing interests

The authors declare no competing interest.
